# Supplementary figures and images for: Presence of small resistant peptides from new in vitro digestion assays detected by liquid chromatography tandem mass spectrometry: An implication of allergenicity prediction of novel proteins?
Source: PLoS One. 2020 Jun 15;15(6):e0233745. doi: 10.1371/journal.pone.0233745 (PMC7295189; doi:10.1371/journal.pone.0233745)

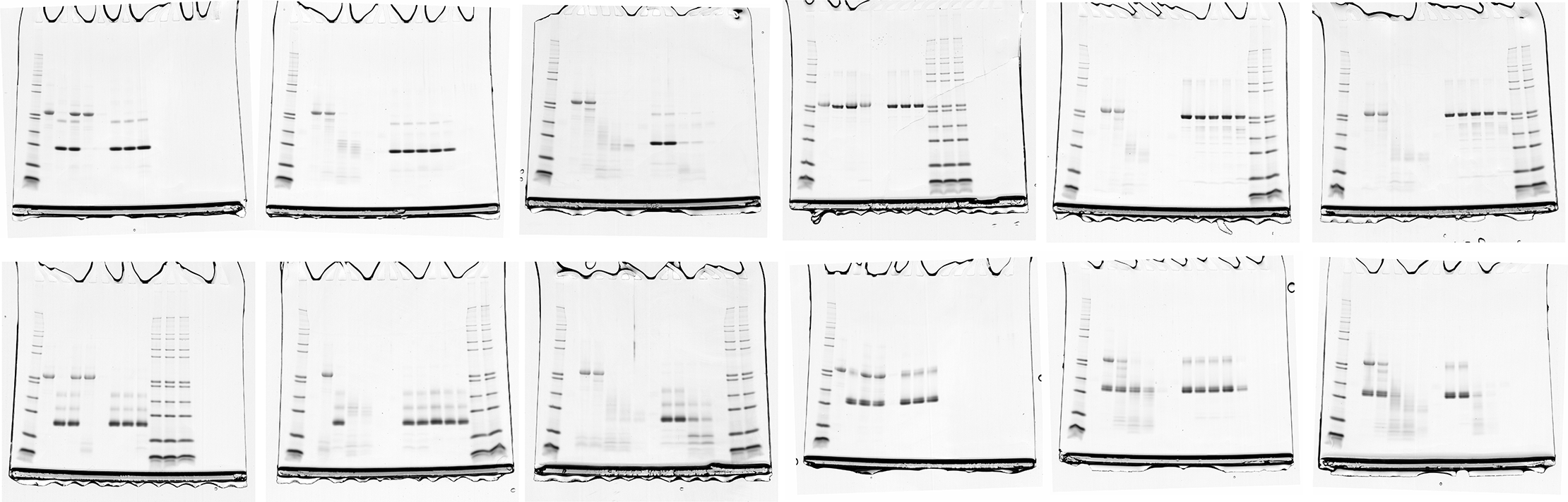

Supplement: S1 Raw Image — (TIF) [file pone.0233745.s004.tif]
