# Supplementary material for: Presence of small resistant peptides from new in vitro digestion assays detected by liquid chromatography tandem mass spectrometry: An implication of allergenicity prediction of novel proteins?
Source: PLoS One. 2020 Jun 15;15(6):e0233745. doi: 10.1371/journal.pone.0233745 (PMC7295189; doi:10.1371/journal.pone.0233745)
Supplement: Attachment — Submitted filename: Response to Reviewers.docx [file pone.0233745.s005.docx]

PONE-D-20-05235
Presence of small resistant peptides from new in vitro digestion assays detected by liquid chromatography-tandem mass spectrometry: an implication of allergenicity prediction of novel proteins?
PLOS ONE

Dear Dr. Maria Gasset

Academic Editor

PLOS ONE

Thank you for your recent letter with reviewer comments. Your recognition that our work is a valid and relevant contribution is much appreciated. All concerns are addressed in blue colored text after each reviewer’s comments in the attached original letter for easy of reading. The revised manuscripts with and without tracked changes have been submitted under file names of “Revised Manuscript with Track Changes” and “Revised Manuscript without Track Changes”, respectively. Files, containing names of “Cover Letter_COI”, original gel images and additional mass spectrometry data for “Supporting Information” have also been uploaded.

Please let me know if there are any questions.

Kind regards,

Rong Wang

Bayer Crop Science

_____________________________________________________________________

PONE-D-20-05235
Presence of small resistant peptides from new in vitro digestion assays detected by liquid chromatography-tandem mass spectrometry: an implication of allergenicity prediction of novel proteins?
PLOS ONE

Dear Dr Ron Wang,

Thank you for submitting your manuscript to PLOS ONE and the time taking its review. The reviewer, who I agree with, has had problems in loading the comments and that is the reason I am copying them. We feel that it has merit but does not fully meet PLOS ONE’s publication criteria as it currently stands. Therefore, we invite you to submit a revised version of the manuscript that addresses the points raised during the review process.

"The study provided by the authors is according to state of the art and provides “real life data” comparing 2 allergens versus 2 non-allergenic proteins using the digestion assays with pepsin and chymotrypsin with and without bile salts following the EFSA Guidance document 2017. In their comparative analysis the authors compare 2 allergens and 2 non-allergenic proteins and follow their degradation via different conditions. This guidance document from EFSA J. 2017 states the refined /extended digestion assays are implemented for a time of 2 years to collect data that either support this part of weight of evidence approach or not – so this is a valuable information in that context.

The study is valid and highly relevant, state of the art, and provides data for the risk assessment discussion while it does not provide novel aspects.

The authors conclude that using different protocols for digestion assays and subsequent LC-MS detection does not provide additional information regarding allergenic risk assessment. Their conclusions is to stay with the established pepsin digestion assay which is sufficient for a weight of evidence approach for safety assessment of potential allergens.

What is missing is a discussion on peptides relevant for celiac disease where a) a library of peptides is available and b) the restriction of HLA types allows a thorough search for respective peptides. Has this been performed – or can this be discussed accordingly? Again this would be of relevance for the EFSA interim phase -see guidance document 2017. The discussion focuses on the enzyme kinetics and optimal conditions – true and relevant however, for physiological conditions  in humans it may be a different scenario – this should be reflected in the discussion. Also the aspect of a potential protective role of phosphatidylcholine for digestion (Ref. Mandalari et al) should be discussed. Furthermore, as presented in the guidance document aspects on celiac disease and the relevant peptides should be included in the discussion.

Minor criticism:

What was the underlying rationale of selecting the non-allergenic proteins"

We would appreciate receiving your revised manuscript by May 15th. When you are ready to submit your revision, log on to <https://www.editorialmanager.com/pone/> and select the 'Submissions Needing Revision' folder to locate your manuscript file.

If you would like to make changes to your financial disclosure, please include your updated statement in your cover letter.

To enhance the reproducibility of your results, we recommend that if applicable you deposit your laboratory protocols in protocols.io, where a protocol can be assigned its own identifier (DOI) such that it can be cited independently in the future. For instructions see: <http://journals.plos.org/plosone/s/submission-guidelines#loc-laboratory-protocols>

Please include the following items when submitting your revised manuscript:

- A rebuttal letter that responds to each point raised by the academic editor and reviewer(s). This letter should be uploaded as separate file and labeled 'Response to Reviewers'.

Response:

The rebuttal letter with a file name of “Response to Reviewers” has been uploaded.

- A marked-up copy of your manuscript that highlights changes made to the original version. This file should be uploaded as separate file and labeled 'Revised Manuscript with Track Changes'.

Response:

A revised manuscript with track changes is uploaded.

- An unmarked version of your revised paper without tracked changes. This file should be uploaded as separate file and labeled 'Manuscript'.

Response:

A revised manuscript without track changes is uploaded.

Please note while forming your response, if your article is accepted, you may have the opportunity to make the peer review history publicly available. The record will include editor decision letters (with reviews) and your responses to reviewer comments. If eligible, we will contact you to opt in or out.

We look forward to receiving your revised manuscript.

Kind regards,

Maria Gasset, Ph.D.
Academic Editor
PLOS ONE


Additional Editor Comments (if provided):

The study provided by the authors is according to state of the art and provides “real life data” comparing 2 allergens versus 2 non-allergenic proteins using the digestion assays with pepsin and chymotrypsin with and without bile salts following the EFSA Guidance document 2017. In their comparative analysis the authors compare 2 allergens and 2 non-allergenic proteins and follow their degradation via different conditions. This guidance document from EFSA J. 2017 states the refined /extended digestion assays are implemented for a time of 2 years to collect data that either support this part of weight of evidence approach or not – so this is a valuable information in that context.
The study is valid and highly relevant, state of the art, and provides data for the risk assessment discussion while it does not provide novel aspects.
The authors conclude that using different protocols for digestion assays and subsequent LC-MS detection does not provide additional information regarding allergenic risk assessment. Their conclusions is to stay with the established pepsin digestion assay which is sufficient for a weight of evidence approach for safety assessment of potential allergens.
What is missing is a discussion on peptides relevant for celiac disease where a) a library of peptides is available and b) the restriction of HLA types allows a thorough search for respective peptides. Has this been performed – or can this be discussed accordingly? Again this would be of relevance for the EFSA interim phase -see guidance document 2017. The discussion focuses on the enzyme kinetics and optimal conditions – true and relevant however, for physiological conditions in humans it may be a different scenario – this should be reflected in the discussion. Also the aspect of a potential protective role of phosphatidylcholine for digestion (Ref. Mandalari et al) should be discussed. Furthermore, as presented in the guidance document aspects on celiac disease and the relevant peptides should be included in the discussion.

Response:

Thank you for your positive feedback. It is very encouraging that our work is recognized as valuable and relevant to the risk assessment discussion. Additional discussion text has been added to the revised manuscript to address the reviewer’s comments.

Per reviewer’s first point, the insightful question aimed the exact essence of the relevance of 9 amino acid detection and is very much appreciated. Our additional discussion on peptide search and its relevance for non-IgE mediated celiac disease assessment is as follows:

Wheat gluten proteins, rich in proline and glutamine, and their allergenic epitopes are well characterized. For celiac disease, the 9 amino acid allergenic peptide has a distinct pattern of Q/E-X1-P-X2 motif, which can be presented by disease associated HLA-DQ (Human Leukocyte Antigen-DQ) molecules and can subsequently be recognized by T cells of celiac disease patients. A list of sequences containing this motif with their degenerated sequences was proposed by EFSA for sequence comparison (1). Such an evaluation is valuable and can inform on potential exposure to gluten or gluten-like peptides. A comparison of the amino acid sequences of all four proteins tested in the present study (i.e. β-Lg, α-La, Hb, and PFK) to the list of peptide sequences was conducted and revealed no relevant matches to known allergenic peptide sequences. Thus, evaluation of a test protein for the presence of allergenic peptide sequences can be done *in silico*, which has been routinely provided as part of the weight of evidence for the safety of newly expressed proteins in transgenic plants since early 2000 (CODEX, 2003). The combination of pepsin resistance analysis and *in silico* sequence homology analysis is sufficiently robust enough to address the question on protein stability against pepsin and sequence similarity to allergens.

Per reviewer’s second point, physiological conditions in humans may indeed be a different scenario than the refined digestion conditions historically used. We pointed out in the Discussion (line 306 in original manuscript) that the physiological conditions of *in vivo* protein digestion are highly complex and constantly changing, such that no single *in vitro* model can fully represent, or simulate, the *in vivo* digestion processes. We also commented in the discussion (line 427 in original manuscript) that the overly simplified physiological digestion conditions through so-called refinement can neither truly reflect physiological conditions nor provide more reliable knowledge about the susceptibility of a NEP in the gastrointestinal tract. The *in vitro* digestibility assessment is useful to assess a protein’s physiochemical property at a define condition and provide attainable snapshot information.

Per reviewer’s third point, there is the aspect of a potential protective role of phosphatidylcholine (PC) for digestion (2). Mandalari *et al* observed that PC had no effect on pepsin digestion while it showed protection of β-Lg under duodenal digestion. Here are a few thoughts on her observations and why bile salts would be a relevant additive for a *in vitro* assay. First, Mandalari *et al* only tested one protein, β-Lg and concluded that PC had a protective role. Second, the critical micelle concentration (CMC) for PC is ~7 μM (2) vs 6 mM for sodium cholate or 16 mM for sodium deoxycholate which are two main components in bile salts. Low concentration of PC at μM are found in human gastric compartment (3). Therefore, the experimental concentration at 3 mM (2) well exceeded the CMC and physiological condition could form protection layers for proteins. Third, proteins in aqueous solution with low pH are expectedly unfolded and hydrolyzed during pepsin digestion while neutral pH and micelle formation could have enough protection on β-Lg during duodenal digestion. Finally, bile salts are major components of bile acid and suitable as a co-cofactor of intestinal digestion.

We agree with reviewer that the relevant peptide should be evaluated rather than focus on peptide detection. This is clarified in the revised manuscript.

Minor criticism:
What was the underlying rationale of selecting the non-allergenic protein

Response:

Hemoglobin (Hb) has been used to assess pepsin activity. We had the familiarity of Hb and studied Hb digested by pepsin at a broad pH range from 1.2 to 6 in a previous published paper (4). phosphofructokinase (PFK) was investigated by Astwood et al as pepsin labile protein (5) and recommended by EFSA as an assay control protein (1). Both Hb and PFK are non-allergens. Two allergens, α-La and β-Lg, a pepsin labile and a pepsin resistant, have been well studies. Both our experience and obtained literature knowledge can help us interpret data. Four proteins provided the representative spectra – digestible vs non-digestible, allergen vs non-allergen.

Journal requirements:

When submitting your revision, we need you to address these additional requirements:

1.    Please ensure that your manuscript meets PLOS ONE's style requirements, including those for file naming. The PLOS ONE style templates can be found at <http://www.plosone.org/attachments/PLOSOne_formatting_sample_main_body.pdf> and <http://www.plosone.org/attachments/PLOSOne_formatting_sample_title_authors_affiliations.pdf>

Response:

Format of the manuscript has been updated to meet PLOS ONE requirement.

2. PLOS ONE now requires that authors provide the original uncropped and unadjusted images underlying all blot or gel results reported in a submission’s figures or Supporting Information files. This policy and the journal’s other requirements for blot/gel reporting and figure preparation are described in detail at <https://journals.plos.org/plosone/s/figures#loc-blot-and-gel-reporting-requirements> and <https://journals.plos.org/plosone/s/figures#loc-preparing-figures-from-image-files>. When you submit your revised manuscript, please ensure that your figures adhere fully to these guidelines and provide the original underlying images for all blot or gel data reported in your submission. See the following link for instructions on providing the original image data: <https://journals.plos.org/plosone/s/figures#loc-original-images-for-blots-and-gels>.

Response:

The original uncropped and unadjusted gel images are uploaded as Supporting Information.

In your cover letter, please note whether your blot/gel image data are in Supporting Information or posted at a public data repository, provide the repository URL if relevant, and provide specific details as to which raw blot/gel images, if any, are not available. Email us at [plosone@plos.org](mailto:plosone@plos.org) if you have any questions.

Response:

A cover letter is updated to indicate original uncropped and unadjusted gel image files are in Supporting Information

3. Thank you for stating the following in the Competing Interests section:

"I have read the journal's policy and the authors of this manuscript have the following competing interest: The authors are employees of Bayer CropScience, a leading manufacturer of crop seeds developed through conventional breeding or biotechnology. The funder had no role in study design, data collection and analysis, decision to publish, or preparation of the manuscript."

 Please confirm that this does not alter your adherence to all PLOS ONE policies on sharing data and materials, by including the following statement: "This does not alter our adherence to  PLOS ONE policies on sharing data and materials.” (as detailed online in our guide for authors [http://journals.plos.org/plosone/s/competing-interests](about:blank)).  If there are restrictions on sharing of data and/or materials, please state these. Please note that we cannot proceed with consideration of your article until this information has been declared.

Please include your updated Competing Interests statement in your cover letter; we will change the online submission form on your behalf.

Response:

The statement that "This does not alter our adherence to  PLOS ONE policies on sharing data and materials” was confirmed and included in the cover letter.

Please know it is PLOS ONE policy for corresponding authors to declare, on behalf of all authors, all potential competing interests for the purposes of transparency. PLOS defines a competing interest as anything that interferes with, or could reasonably be perceived as interfering with, the full and objective presentation, peer review, editorial decision-making, or publication of research or non-research articles submitted to one of the journals. Competing interests can be financial or non-financial, professional, or personal. Competing interests can arise in relationship to an organization or another person. Please follow this link to our website for more details on competing interests: [http://journals.plos.org/plosone/s/competing-interests](about:blank)

Response:

I, the corresponding authors, declared, on behalf of all authors, all potential competing interests for the purposes of transparency

4. We note that you have included the phrase “data not shown” in your manuscript. Unfortunately, this does not meet our data sharing requirements. PLOS does not permit references to inaccessible data. We require that authors provide all relevant data within the paper, Supporting Information files, or in an acceptable, public repository. Please add a citation to support this phrase or upload the data that corresponds with these findings to a stable repository (such as Figshare or Dryad) and provide and URLs, DOIs, or accession numbers that may be used to access these data. Or, if the data are not a core part of the research being presented in your study, we ask that you remove the phrase that refers to these data.

Response:

Clarification was made to replace “data not shown”. Additional data with file name Supp_Table2.xlxs that contain number of peptides and peptide sequences was uploaded as Supporting Information

References:

1.	Naegeli H, Birch A, Casacuberta J, De Schrijver A, Gralak M, Guerche P, et al. Guidance on allergenicity assessment of genetically modified plants. EFSA Journal. 2017;15.

2.	Mandalari G, Mackie AM, Rigby NM, Wickham MS, Mills EN. Physiological phosphatidylcholine protects bovine beta-lactoglobulin from simulated gastrointestinal proteolysis. Molecular nutrition & food research. 2009;53 Suppl 1:S131-9.

3.	Minekus M, Alminger M, Alvito P, Ballance S, Bohn T, Bourlieu C, et al. A standardised static in vitro digestion method suitable for food - an international consensus. Food & Function. 2014;5(6):1113-24.

4.	Wang R, Edrington TC, Storrs SB, Crowley KS, Ward JM, Lee TC, et al. Analyzing pepsin degradation assay conditions used for allergenicity assessments to ensure that pepsin susceptible and pepsin resistant dietary proteins are distinguishable. PloS one. 2017;12(2):e0171926.

5.	Astwood JD, Leach JN, Fuchs RL. Stability of food allergens to digestion in vitro. Nature biotechnology. 1996;14(10):1269-73.
